# E-Cadherin Expression Distinguishes Mouse from Human Hematopoiesis in the Basophil and Erythroid Lineages

**DOI:** 10.3390/biom12111706

**Published:** 2022-11-17

**Authors:** Rosa A. Krimpenfort, Felix M. Behr, Marja Nieuwland, Iris de Rink, Ron Kerkhoven, Marieke von Lindern, Micha Nethe

**Affiliations:** 1 Sanquin Research, Landsteiner Laboratory, Academic Medical Centre, Department of Hematopoiesis, University of Amsterdam, 1066 CX Amsterdam, The Netherlands; 2 Genomics Core Facility, Netherlands Cancer Institute, 1006 BE Amsterdam, The Netherlands

**Keywords:** E-cadherin, erythropoiesis, hematopoiesis, basophil, erythroblast

## Abstract

E-cadherin is a key regulator of epithelial cell–cell adhesion, the loss of which accelerates tumor growth and invasion. E-cadherin is also expressed in hematopoietic cells as well as epithelia. The function of hematopoietic E-cadherin is, however, mostly elusive. In this study, we explored the validity of mouse models to functionally investigate the role of hematopoietic E-cadherin in human hematopoiesis. We generated a hematopoietic-specific E-cadherin knockout mouse model. In mice, hematopoietic E-cadherin is predominantly expressed within the basophil lineage, the expression of which is dispensable for the generation of basophils. However, neither E-cadherin mRNA nor protein were detected in human basophils. In contrast, human hematopoietic E-cadherin marks the erythroid lineage. E-cadherin expression in hematopoiesis thereby revealed striking evolutionary differences between the basophil and erythroid cell lineage in humans and mice. This is remarkable as E-cadherin expression in epithelia is highly conserved among vertebrates including humans and mice. Our study therefore revealed that the mouse does not represent a suitable model to study the function of E-cadherin in human hematopoiesis and an alternative means to study the role of E-cadherin in human erythropoiesis needs to be developed.

## 1. Introduction

E-cadherin is a transmembrane protein and a master regulator of cell adherens junctions, through which it controls cell–cell adhesion. Epithelial cell–cell adhesion controls organ function, prevents tissue infection and suppresses malignancy formation. E-cadherin expression, however, can be detected in a wide variety of cells including hematopoietic progenitor cells (HPCs). Adherence junctions are formed by multimeric homophilic E-cadherin protein interactions at the interface between neighboring cells [1,2]. Intracellularly, E-cadherin binds signaling molecules α- and β-catenin which connect the actomyosin cytoskeleton with the site of adhesion [3]. Cell adherens junctions thereby act as molecular bridges that are able to transmit mechanical forces generated by neighboring cells (e.g., by cell migration, proliferation or apoptosis [4,5,6,7]. Mechanical activation of E-cadherin/β-catenin signaling accordingly promotes cell proliferation, which underlies the regulation of E-cadherin in tissue homeostasis [6,7]. The importance of E-cadherin signaling during epithelial development is signified in E-cadherin-null mutant mice, which are embryonic lethal due to failure of trophectoderm epithelium formation [8]. E-cadherin is also a well-established tumor suppressor as demonstrated in mouse models with tissue-specific conditional inactivation of E-cadherin, which revealed impaired intestinal, skin and mammary gland formation and promoted tumor development and metastasis [9,10,11,12,13]. Moreover, *CDH1* (encoding E-cadherin) inactivating mutations or promoter hypermethylation in humans are associated with the development of gastric, prostate, hepatocellular, breast and esophageal carcinomas [14]. Overall, E-cadherin [6,7] exerts a profound control over epithelial cell behavior and acts as an important tumor suppressor.

In addition to its uses in the epithelia, E-cadherin expression can be used to discriminate hematopoietic neoplasms and is used as a marker of pure erythroid leukemia [15,16]. Downregulation of E-cadherin by promoter methylation in acute myeloid leukemia (AML) has also been indicated to reflect poor disease outcome [17,18,19]. Whether hematopoietic E-cadherin may act as a tumor suppressor in bone marrow (BM), as seen in epithelia, remains, however, to be defined. In hematopoiesis, E-cadherin has been found to be expressed by erythroid precursors in human BM and demonstrated to mark the basophil/mast cell lineage in mice [20,21,22]. However, the role of E-cadherin in erythropoiesis and basophil development remains elusive. In this study, we investigate E-cadherin expression in mouse hematopoiesis, in comparison to human, in order to define whether mice can be used as a model to investigate the function of hematopoietic E-cadherin in humans.

## 2. Methods

### 2.1. Generation of Mice

*Vav-iCre* transgenic mice were obtained from the Jackson Laboratory (B6.Cg-Tg(*Vav1-Cre*)A2Kio/J, stock no. 008610] and crossed with *Cdh1^F/F^;mTmG* mice as previously described [10] to generate *Vav-iCre;Cdh1^F/F^;mTmG* mice. Genotyping was performed using the following primers: (*Vav-iCre*;5′–AGATGCCAGGACATCAGGAACCTG–3′ and 5′-ATCAGCCACACCAGACACAGAGATC-3′, *Cdh1*;5′-TCAATCTCAGAGCCCCACCTA-3′ and 5′-CCTGCCATGATTGTCATGGAC-3′, *mTmG*;5′-CGAGGCGGATCACAAGCAATA-3′ and 5′- TCAATGGGCGGGGGTCGTT-3′. All mice were maintained in the animal facility of the Netherlands Cancer Institute (NKI). Animal experiments were conducted according to institutional and national guidelines.

### 2.2. Human PBMC Isolation

Human material was obtained after informed consent was given in accordance with the Declaration of Helsinki, the Dutch National and Sanquin Internal Ethic Boards. BM was aspirated from patients undergoing cardiac surgery (permit MEC 04/042, No. 04.17.370; AMC, Amsterdam, The Netherlands). Human peripheral blood mononuclear cells (PBMCs) were purified by density separation using Ficoll (GEHealthcare, 17-1440-03), following the manufacturer’s protocol. For hematopoietic stem and progenitor cell (HSPC) isolation, mobilized peripheral blood (MPB) was obtained by leukapheresis. HSPCs were isolated using CD34+ MACS beads (Miltenyi, 130-046-703) according to the manufacturer’s protocol and cryopreserved in IMDM, 20% FCS; 5% DMSO as previously described [23].

Mouse tissue preparation mouse BM cells were obtained by crushing the femur and tibia, and a 70 µM cell strainer was used to filter the suspension. Spleen cells were directly mashed through a 70 µM cell strainer. Consequently, the cell strainer was flushed with PBS containing 1% FCS. Cells were kept at 4 °C at all times. Blood was collected through heart puncture in a heparin tube. Whole blood was incubated with erythrocyte lysis buffer (0.155 M NH_4_Cl; 10 mM KHCO_3_; 1 mM EDTA; pH 7.2–7.6) before antibody staining for flow cytometry. Lung tissue was cut into pieces and enzymatically digested for 30 min at 37 °C with 750 U/mL Collagenase Type I (Worthington, LS004194) and 0.31 mg/mL DNase I (Roche, 10104159001). Single cells were separated over a Percoll (GE Healthcare, 17-0891-01) density gradient following the manufacturer’s protocol before proceeding with hematopoietic fraction for flow cytometry. Peritoneal cells were obtained by flushing the peritoneal cavity with PBS. Blood values were measured using a Scil Vet abc Plus machine (Horiba Medical, Montpellier, France) using the pre-installed mouse research program.

### 2.3. LCMV Infections

In order to induce a representative multilineage challenge including the red cell lineage, mice were infected intraperitoneally with 1 × 105 plaque-forming units (PFU) of LCMV Armstrong obtained from EVAg, European Virus Archive. After 8 days, the mice were sacrificed and their organs were processed according to the methods above.

### 2.4. Immunohistochemistry

Mouse BM was formalin-fixed in 10% neutral buffered formalin for 48 h, embedded in paraffin, sectioned and stained with hematoxylin and eosin (H&E). Immunohistochemistry (IHC) was performed as previously described [10]. All slides were digitally processed using the Aperio ScanScope (Aperio, Vista, CA, USA) and captured using ImageScope software version 12.0.0 (Aperio).

### 2.5. Flow Cytometry

Cells were incubated with antibodies for 30 min at 4 °C as listed in Supplementary Table S1 (for mouse tissue) and Table S2 (for human tissue). When specified, human PBMCs were incubated with 100 µg/mL Nanogam, broad spectrum human normal immunoglobulin (Prothya Biosolutions, Amsterdam, The Netherlands) for FcR-blocking as control for isotype staining. Dead cells were excluded by using the fixable near-IR live/dead staining kit (Thermo Fisher Scientific (Waltham, MA, USA), L10119). A Foxp3/Transcription Factor Staining Buffer Set (eBioscience (San Diego, CA, USA), 00-5523-00) was used according to the manufacturer’s specifications for intracellular staining. Flow cytometry was performed on an FACS Symphony (BD Biosciences, Haryana, India), and data was analyzed using FlowJo V10 (Tree star) software. Cell sorting was performed on an FACS Aria III (BD Biosciences), and on-site analysis was performed using BD FACSDiva 8.0.1. software.

### 2.6. Cytospins

Mouse E-cadherin^+^;IgE^+^ cells were spun down at 800 rpm on an object glass using a Shandon Cytospin II (Thermo Scientific), air dried and fixed in methanol for 5 min. Samples were stained with May–Grünwalds eosine-methylene blue solution (Merck, Rahway, NJ, USA) for 10 min and Giemsa dilution for 20 min.

### 2.7. Hematopoietic Progenitor Colony Formation Assay

For progenitor analysis by E-cadherin cells, 2500 mouse bone marrow E-cadherin^+^;cKIT^+^, Ecadherin^+^;cKIT^−^ and LK cells were sorted and plated in duplicate in Methocult (Stemcell Technologies (Vancouver, BC, Canada), GF M3434). Colonies formed were characterized and counted 5–14 days after plating.

### 2.8. Single Cell RNA Sequencing

E-cadherin^+^;cKIT^+^ and Ecadherin^+^;cKIT^−^ cells were sorted and used at a 2x single-cell suspension at final conc. 1000 cells/µL in 1 × PBS containing 0.04% weight/volume BSA (400 µg/mL). Cells were loaded into a 10X Genomics Chromium Controller for capture and barcoding following the 10X Genomics Protocol (Chromium Single Cell 3′ Reagent Kits v3, CG000183 Rev A), as described by the manufacturer (10X Genomics). See supplementary information for more details.

The molarity of the Single Cell 3′Gene Expression libraries were quantified on a 2100 Bioanalyzer Instrument following the Agilent Technologies Protocol (Agilent DNA 7500 kit, G2938-90024), as described by the manufacturer (Agilent Technologies, Santa Clara, CA, USA). The Single Cell 3′ Gene Expression libraries were diluted to 10 nM and pooled to create one Illumina sequencing library pool. The molarity of the Illumina sequencing library pool was quantified by qPCR following the Roche Protocol (KAPA Library Quantification Kit Illumina^®^ Platforms, KR0405), as described by the manufacturer. Single-cell RNA sequencing was performed on the Illumina Nextseq 550 system (Illumina). The samples were sequenced on a NextSeq High with a base composition of 28-8-56. Sample 1 had a total of 265 million reads and sample 2 had a total of 72 million reads. Downstream analysis was performed using Cellranger version 3.0.2 with mm10 as a reference genome. In total, 6632 and 3745 cells were picked up for sample 1 and 2 with a median of 3.3 K and 1.7 K detected genes per cell, an average of 40 K and 19 K reads per cell, and median UMI counts per cell of 13 K and 4.5 K, respectively. The output data of Cellranger has been used for further in-depth analysis. In-depth analysis was performed within R using the Seurat package (version 3).

Genes that were expressed in less than 40 cells were removed from the individual datasets which resulted in 6632 and 3745 cells. Individual datasets have also been filtered for the highest and lowest 1% number of genes and highest 1% number of RNA counts in order to remove outliers from the dataset. A total of 12,395 and 10,412 genes were detected in these samples. After these filtering steps, the datasets were merged, which resulted in a total of 10,141 (6279 and 3662) cells and 12,462 genes. Cells have not been filtered for mitochondrial expression because the expression levels of mitochondrial genes were very low. Normalization, scaling and PCA dimensionality reduction of the data have been performed using standard Seurat parameters. To identify clusters, the TSNE algorithm for dimensionality reduction has been used. t-SNE clusters were automatically detected by Seurat using the FindNeighors/FindClusters functions with 15 dimensions and the run t-SNE function with a resolution of 1.

The Series record can be accessed at https://www.ncbi.nlm.nih.gov/geo/query/acc.cgi?acc=GSE211102 (accessed on 15 November 2022).

### 2.9. Statistics

GraphPad Prism 6 software was used to apply a standard two-sided Student’s *t*-test (paired) for pairwise comparisons. *p* < 0.05 values were considered statistically significant.

## 3. Results

### 3.1. E-cadherin Is Predominantly Expressed by Basophil/Mast Cell Precursors in Mouse BM

**Figure 1:**
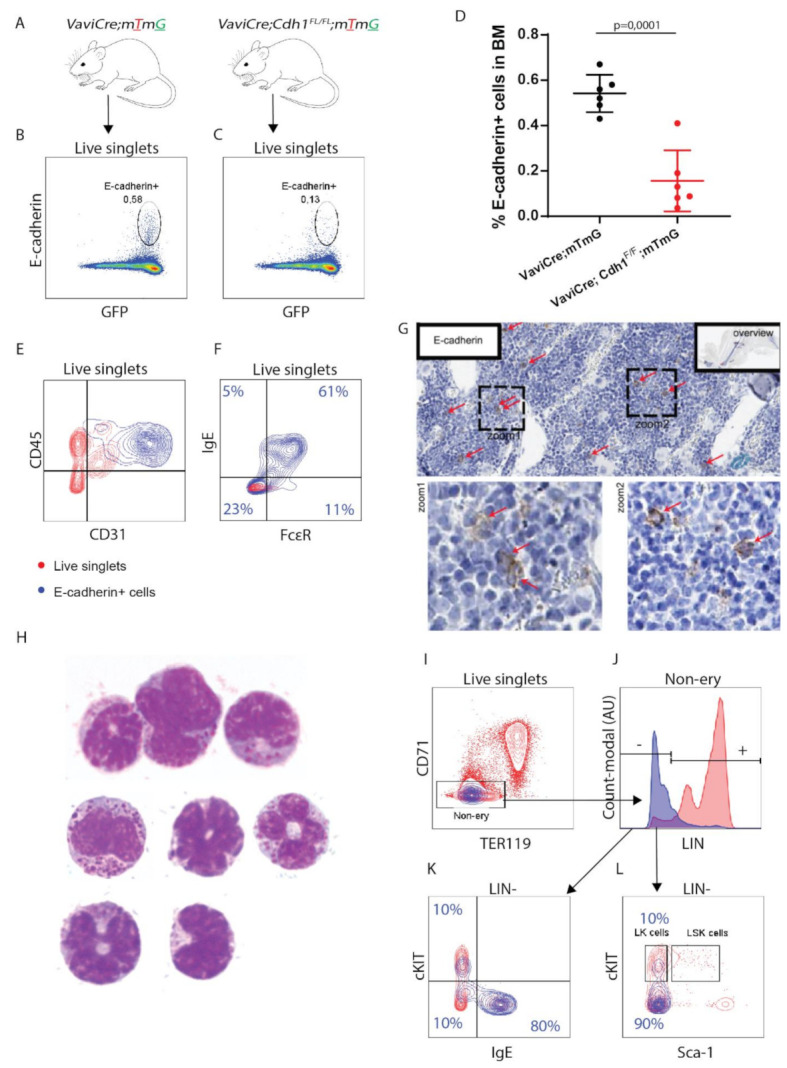
E-cadherin is sporadically expressed in the mouse hematopoietic cell lineage. (**A**) Schematic illustration of hematopoietic-specific E-cadherin (*Cdh1*) KO and control mouse models. Vav-iCre mice, which specifically express Cre recombinase in the hematopoietic cell lineage, were crossed with *Cdh1^F/F^;mTmG* mice to inactivate *Cdh1* in hematopoiesis. Cre activity was monitored by GFP expression as induced upon recombination of the mTmG reporter. *Vav-iCre;mTmG* mice were generated in parallel and used as control animals. (**B**) Representative FACS analysis of E-cadherin cell-surface expression by bone marrow (BM) cells derived from *VaviCre;mTmG* mice. Cre activity was monitored in parallel by measuring GFP fluorescence (*n* = 6) (gating strategy provided in Supplementary Figure S1A–D). (**C**) Representative FACS analysis to examine E-cadherin cell-surface expression within the GFP-marked hematopoietic cell lineage in BM of *VaviCre;Cdh1^F/F^;mTmG* mice as defined by FACS (*n* = 6). (**D**) Quantification of E-cadherin surface expression in hematopoiesis in *VaviCre;mTmG* and *VaviCre;Cdh1^F/F^;mTmG* mice. (**E**) Characterization of E-cadherin^+^ (in blue) BM cells (E-cadherin^-^ in red) within CD45^+^ CD31^+/−^ (hematopoietic) and CD45^−^ CD31^+^ (stromal/endothelial) compartment by FACS (*n* = 3). (**F**) Representative FACS analysis of E-cadherin cell-surface expression (in blue, E-cadherin^-^ in red) on basophil/mast cell lineage (IgE^+^ FCεR^+^ B220^−^) by FACS. (*n* = 6) (**G**) Immunohistochemical staining for E-cadherin (brown color, indicated by arrow) on mouse BM sections. (**H**) Representative cytospins of E-cadherin^+^ IgE^+^ mouse BM cells stained with May–Grünwald–Giemsa. (**I**) Representative FACS analysis of E-cadherin cell-surface expression (blue) on (CD71^+^ TER119^−^) proerythroblasts and (CD71^+^ TER119^+^) erythroblasts (E-cadherin in red^−^, *n* = 3). (**J**) Representative FACS analysis of E-cadherin (blue) cell-surface coexpression with defined lineage markers (LIN: CD4, CD8, B220, CD11b, Ly6G/C) (E-cadherin^−^ in red, *n* = 3). (**K**) E-cadherin cell-surface expression (blue) defined within (IgE^+^) basophils/mast cell precursors and (cKIT^+^) hematopoietic progenitor populations by FACS (E-cadherin^−^ in red, *n* = 3). (**L**) Characterization of E-cadherin^+^ (in blue) BM cells (E-cadherin^−^ in red) within (LIN^-^cKIT^+^) HPCs and (Lin^−^ Sca^+^ cKIT^+^) HSPCs by FACS (*n* = 3).

To define the role of E-cadherin in the HSPC lineage of mice, we generated *Vav-iCre;Cdh1^F/F^:mTmG* mice by crossing the previously described *V*av-iCre** transgenic mice with *Cdh1^F/F^;mTmG* mice (Figure 1A) [10,24]. *Vav-iCre* was used to induce somatic inactivation of floxed *Cdh1 expression* within the hematopoietic cell lineage. Expression of Cre can accordingly be defined by monitoring GFP fluorescence, which is induced upon Cre-driven recombination of the inserted mTmG reporter gene [24,25]. *Vav-iCre;mTmG* mice were developed as control animals and used to characterize E-cadherin protein expression in the hematopoietic cell lineage.

As E-cadherin predominantly localizes at the plasma membrane, we used flow cytometry to examine the distribution of E-cadherin within the hematopoietic cell lineage in BM of *Vav-iCre;mTmG* mice. Hematopoietic Cre recombinase activity was verified by correlation of GFP-expression with CD45 (Supplementary Figure S1E). E-cadherin expression was detected in ~0.6% of GFP^+^ hematopoietic cells, compared to a background signal of ~0.1% in the *Vavi-Cre;Cdh1^F/F^;mTmG* and *Vavi-Cre;;mTmG* mouse measured with isotype control antibody (Figure 1B–D, Supplementary Figure S1A–D). Hereby we confirm the specificity of E-cadherin detection and suggest successful KO of *Cdh1* expression (Figure 1C,D). Remarkably, as we included CD31 (encoding PECAM-1) staining to define the endothelial compartment (CD45^−^ CD31^+^), we noticed that E-cadherin^+^ hematopoietic cells could be distinguished by high expression of CD31 within CD45^+^ marked hematopoietic compartment (Figure 1E). In line with the observed accumulation of *Cdh1* mRNA within the basophil/mast cell precursor lineage in mouse BM, approximately 60–80% of E-cadherin^+^ hematopoietic cells reflected a basophil/mast cell signature, as defined by cell-surface protein expression of IgE and FcεR (Figure 1F) [21]. E-cadherin thereby marks the entire population of basophil and mast precursors (CD45^+^ IgE^+^ CD45^-^ KIT^-^) in mouse BM. The low percentage of E-cadherin^+^ hematopoietic cells in mouse BM, as detected by flow cytometry, was consistent with the sporadic detection of E-cadherin-expressing cells in mouse BM sections by immunohistochemistry (IHC; Figure 1G). Additionally, E-cadherin is not coexpressed with lymphoid (CD3, CD4, CD8, B220), myeloid (Gr-1, CD11b) or erythroid (CD71, Ter119) lineage (LIN) markers (Figure 1G–J). In contrast, all cells in our basophil gate (IgE^+^ LIN^−^) express cell-surface E-cadherin (Supplementary Figure S1F,G). Cytospins of E-cadherin^+^ IgE^+^ mouse BM cells show, as previously described for mouse BM and blood-derived basophils, polymorphic nuclei and dark granules, confirming their basophilic character (Figure 1H) [26]. The remaining fraction of E-cadherin^+^ cells (~20% corresponding to 0.1% of total hematopoietic BM cells), however, did not reflect a basophil-defined cell-surface signature but expressed intermediate- to high-cell-surface expression of cKIT (Figure 1K). These cells comprise a fraction of progenitor cell-enriched LIN^-^ KIT^+^ (LK) population and therefore may have progenitor potential (Figure 1L). Notably, the low number of detected E-cadherin^+^ hematopoietic cells in mouse BM is also in line with the predominant detection of *Cdh1* mRNA expression in the rare population of basophil/mast cell progenitors in mouse BM. Taken together, these results confirm that E-cadherin is predominantly expressed by the basophil/mast cell lineage in mouse BM. Additionally, we found that E-cadherin may also be expressed by more immature HPCs.

E-cadherin expressing HPCs in mice are not fully committed to the basophil/mast cell lineage. 

**Figure 2:**
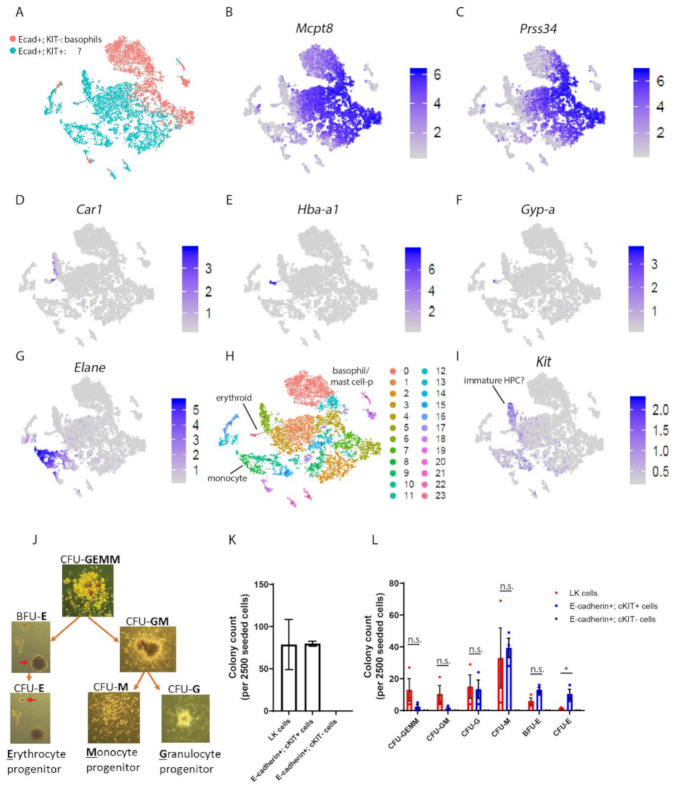
E-cadherin marks late hematopoietic progenitors and basophil/mast cell precursors in the hematopoietic cell lineage of mouse BM (**A**) Single-cell RNA-sequencing from sorted E-cadherin^+^cKIT^+^ (blue) and E-cadherin^+^cKIT^−^ (pink) bone marrow (BM) cells derived from *VaviCre; mTmG* mice. Out of 10377 cells, sorted from 3 mice, 10,141 cells were processed after filtering for t-SNE clustering by Seurat. (**B**) Expression quantiles of genes marking the basophil/mast cell lineage; *Mcpt8*; (**C**) *Prss34*; erythroid; (**D**) *Car1*, (**E**) *Hba-A1*, (**F**) *GYPA*, monocyte; (**G**) *Elane*, bars depict unique molecular identifier (UMI) distribution of these genes based on scaled data across E-cadherin^+^;KIT^+^ (blue) and E-cadherin^+^ cKIT^−^ (pink) BM cells. (**H**) t-SNE plot colored by Seurat t-SNE clusters. In total, 24 clusters have been found. (**I**) Expression quantiles of cKIT bars depict UMI distribution of these genes across E-cadherin^+^cKIT^+^ (blue) and E-cadherin^+^cKIT^−^ (pink) BM cells. (**J**) Colonies formed by E-cadherin^+^ cKIT^+^ cells in methylcellulose-based media. (**K**) Quantification of colonies formed by Lin^-^KIT^+^, E-cadherin^+^cKIT^+^ and E-cadherin^+^cKIT^−^, BM cells as performed by three independent experiments. (**L**) Quantification of colony subtypes derived from LK-cells, E-cadherin^+^cKIT^+^ and E-cadherin^+^cKIT^−^ cells.

To define the relationship between E-cadherin^+^cKIT^+^ hematopoietic cells and E-cadherin^+^cKIT^−^ defined basophil/mast cell precursors, we sorted both populations from the BM of wild-type mice and performed single-cell (sc) RNA-seq analysis (Supplementary Figure S2A). The obtained two-dimensional representation revealed that E-cadherin^+^ basophil/mast precursors clustered as one major branch, which separates from the E-cadherin^+^cKIT^+^ cellular compartment (Figure 2A). A large fraction of E-cadherin^+^cKIT^+^ cells were found to coexpress *Mcpt8* and *Prss34* (Figure 2B,C). Prss34 is predominantly expressed by basophils and at low levels by mast cells in the peritoneum [27]. Additionally, *Mcpt8* is used to distinguish the basophil lineage, as it is not expressed by mast cells in mice, although its expression is also detected at low levels in granulocyte/macrophage progenitors (GMPs) [27,28,29,30,31]. Altogether, these findings indicate that the majority of E-cadherin^+^cKIT^+^ hematopoietic cells preferentially act as precursor cells of the basophil/mast cell lineage.

However, the presence of two additional smaller branches suggests that E-cadherin^+^cKIT^+^ cells are not fully committed to the basophil lineage, as these branches express erythroid (*Car1, Hba-a1, Gypa*, and *Tfrc* Figure 2D–F, Supplementary Figure S2F) and monocyte (*Elane*) cell lineage markers (Figure 2G). Additionally, we identified a cell cluster with high-cKIT expression that lacked distinguished lineage markers (cluster #6) and therefore may resemble non-committed early HPCs (Figure 2I).

To test E-cadherin^+^;cKIT^+^ progenitor capacity, we performed colony formation assays with sorted E-cadherin^+^.cKIT^+^ BM-derived cells (Supplementary Figure S2B,C) [21]. We compared the progenitor capacity of E-cadherin^+^cKIT^+^ cells with cell outgrowth of LK-marked HPCs (Supplementary Figure S2D,E). E-cadherin^+^cKIT^+^ hematopoietic cells mostly represent granulocyte colony forming units (CFU-G) and monocyte (CFU-M) progenitors (Figure 2J–L). E-cadherin^+^cKIT^+^ cells, however, also include erythroid progenitors, as revealed by the formation of BFU-E (burst-forming units) and CFU-Es. Additionally, E-cadherin^+^ cKIT^+^ hematopoietic cells were found to comprise a small fraction of multipotent hematopoietic progenitors, as revealed by the generation of CFU-GEMMs (Granulocyte-Erythroid-Monocyte-Megakaryocyte progenitors) and CFU-GMs (Granulocyte-Macrophage progenitors). The latter is also in line with the identified cluster of immature E-cadherin^+^ cKIT^+^ (#6) by scRNAseq (Figure 2I). E-cadherin^+^cKIT^-^-marked basophils, that were taken along as negative controls, lacked colony-forming potential (Figure 2K,L). Overall, these findings revealed that E-cadherin mostly marks late myeloid HPCs in mouse BM, which predominantly, but not exclusively, mark progenitors of the basophil lineage.

### 3.2. E-cadherin Is Dispensable for Basophil Formation in Mice

**Figure 3:**
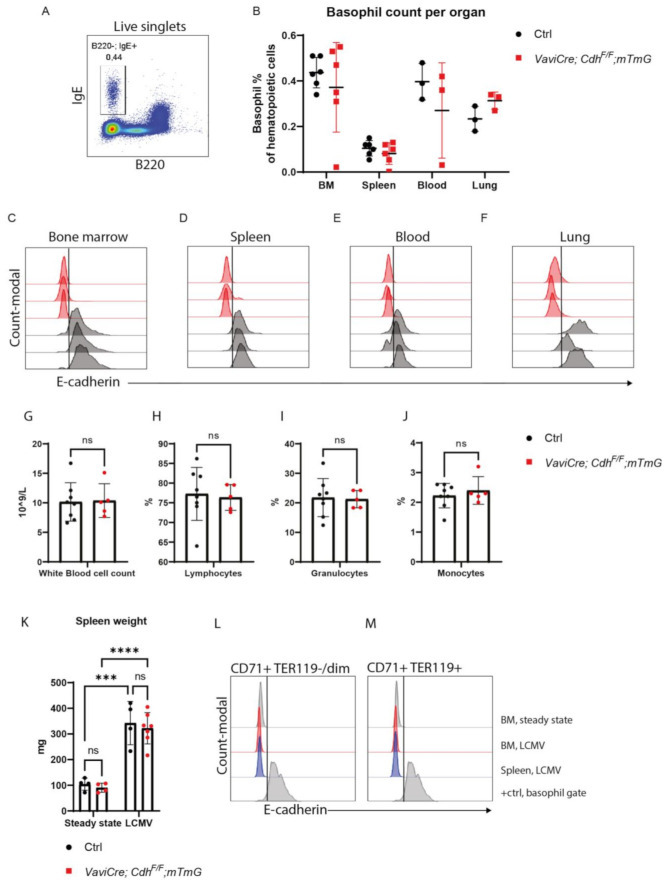
E-cadherin is dispensable for the generation of the basophil and erythroid lineage in mice (**A**) Representative gating strategy of mouse basophil population within live singlets. (**B**) Quantification of the percentage of basophils in hematopoietic cells isolated from *VaviCre;mTmG* (control; black dotted) and *VaviCre;Cdh1^F/F^;mTmG* (red dotted) bone marrow (BM) (*n* = 6 animals), spleen (*n* = 6 animals), blood (*n* = 3 animals) and lung (*n* = 3 animals) (gating strategy provided in Supplementary Figure S3A–E). Cell-surface expression of E-cadherin by basophils in (**C**) BM, (**D**) spleen, (**E**) blood and (**F**) lung, defined by FACS in *VaviCre;mTmG* (grey, *n* = 3 animals) and *VaviCre*;*Cdh1^F/F^;mTmG* mice (pink, *n* = 3 animals). Results are depicted in histograms (X-axis; E-cadherin, Y-axis; Count-modal (A.U)). (**G**) Quantification of erythroblasts (CD71^+^; TER119^+^) in BM and (**H**) spleen in *VaviCre;mTmG* (black dots, *n* = 14 animals) and *VaviCre*;*Cdh1^F/F^;mTmG* (red dots, *n* = 14 animals) mice as defined by FACS (gating strategy provided in Supplementary Figure S3E,F). (**G**) White blood cell counts per L whole blood, subdivided in lymphocyte (**H**), granulocyte (**I**) and monocyte (**J**) percentages measured in whole blood of *VaviCre;mTmG* (control; black dotted *n* = 8) and *VaviCre*;*Cdh1^F/F^;mTmG* (red dotted, *n* = 5) mice. (**K**) Spleen weight (mg) isolated from *VaviCre*;*mTmG* (black dots, *n* = 4; *n* = 4 animals) and *VaviCre*;*Cdh1^F/F^;mTmG* mice (red dots, *n* = 4; *n* = 7 animals) in steady state and during anemia of inflammation. Wild-type erythroid progenitors do not upregulate E-cadherin expression upon anemia of inflammation in the CD71^+^ TER119^−^ (**L**), nor in the CD71^+^ TER119^+^ stage (**M**); *** *p* < 0.001; **** *p* < 0.0001.

Since E-cadherin mostly marks basophil precursors in mouse BM, we next defined whether E-cadherin expression is required for the generation of basophils. We therefore measured the percentage of basophil precursors in BM hematopoietic cells as well as matured basophils in circulation and peripheral organs such as the spleen and lungs in *VaviCre;Cdh1^F/F^;mTmG* mice by flow cytometry (Figure 3A,B). Basophils (IgE^+^ B220^-^) from these organs were confirmed to be negative for cKIT cell-surface expression, which discriminates basophils from mast cells (Supplementary Figure S3A–D). Basophils in *VaviCre;Cdh1^F/F^;mTmG* mice completely lacked cell-surface expression of E-cadherin, suggesting a complete KO of E-cadherin in the basophil lineage, as compared to control mice (Figure 3C–F). However, basophil precursor numbers in BM (~0.4%) as well as the generation of maturated basophils in the spleen (~0.1%), blood (~0.3%) and lungs (~0.3%) of *VaviCre;Cdh1^F/F^;mTmG* mice were not hampered as compared to control animals (Figure 3B). E-cadherin is therefore dispensable for the formation and circulation of basophil/mast cell precursors in mice. Furthermore, white blood cell counts, and the percentages of lymphocytes, monocytes and granulocytes did not differ between control *VaviCre;mTmG* and *VaviCre;Cdh1^F/F^;mTmG* mice (Figure 3G–J).

As a small fraction of E-cadherin^+^ cKIT^+^ cells displayed expression of erythroid transcription markers (Figure 2D–F), as well as erythroid progenitor potential in colony formation assays (Figure 2J–L), we additionally examined erythropoiesis in *VaviCre;Cdh1^F/F^;mTmG* mice to address the control of E-cadherin over mouse erythropoiesis. Loss of hematopoietic E-cadherin, however, does not alter (CD71^+^Ter119^+^) erythroblasts percentage in BM (Supplementary Figure S3G). In mice, the spleen is an important site for extramedullary regulation of erythropoiesis; erythropoiesis in the spleen was also examined. However, hematopoietic-specific loss of E-cadherin did not alter erythroblast percentage in the spleen (Supplementary Figure S3H) [32]. Additionally, as also specified in Figure 1I, there was no E-cadherin cell-surface expression detected in the erythroid lineage in BM or the spleen (Supplementary Figure S3I,J).

As well as steady-state conditions, we investigated the erythroid response to anemia, stress erythropoiesis. We induced anemia by LCMV infection, causing hemolysis and/or INF-γ mediated inflammation [33,34].

Eight days after LCMV infection, red blood cell (RBC) values significantly decreased (Supplementary Figure S3L–N); whereas, spleen weight strongly increased (>300%) (reflecting stress erythropoiesis) in both the control *VaviCre;mTmG* and *VaviCre*;*Cdh1^F/F^;*mTmG mice. Herein, no significant differences were observed between the two groups (Figure 3K, Supplementary Figure S3L–N). Importantly, E-cadherin surface expression was also not upregulated during stress erythropoiesis in (CD71^+^TER119^−^) proerythroblasts or (CD71^+^ TER119^+^) erythroblasts in control *VaviCre;mTmG* mice (Figure 3L,M). Overall, E-cadherin in HPCs in mouse BM is not required for basophil or erythroid outgrowth in mice.

### 3.3. Cdh1 Expression Distinguishes Human from Mouse Hematopoiesis

**Figure 4:**
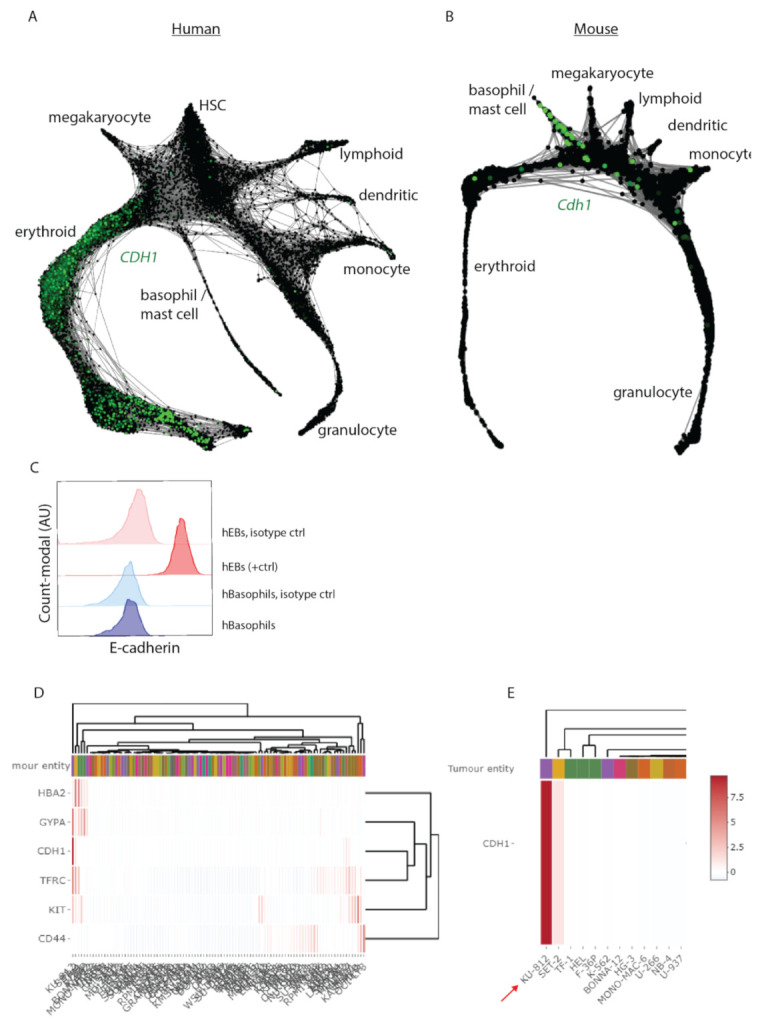
Cdh1 mRNA expression is not conserved between human and mouse hematopoiesis within the erythroid and basophil lineages. (**A**) A previously published high-resolution single-cell RNA-seq dataset of human (Lin^-^CD34^+^CD164^+^) hematopoietic stem and progenitor cells (HSPCs) derived from healthy human donors, resembling all lineage cell fates of HSPCs, data mined for the distribution of *CDH1* in green (encoding E-cadherin) in human and in (**B**) a mouse single-cell RNA-seq data-set, which reflects all principle lineage of the mouse (KIT^+^) HSPCs, reflecting expression of *Cdh1* in green. (**C**) FACS analysis of E-cadherin cell-surface expression by human basophils as compared to human erythroblasts (*n* = 3) depicted in histograms. (**D**) Gene expression analysis of progenitor/erythroid genes *KIT*, *CD44*, *HBA2*, *GYPA*, *TFRC*, and *CDH11* in human leukemic cell lines. (**E**) Zoom into human leukemic cell line KU-812 that expresses *CDH1*.

We here identified that E-cadherin mostly marks the mast/basophil lineage in mice. In contrast, E-cadherin has been reported to mainly mark erythroblasts in human BM [21,22]. This poses the question of to what extent E-cadherin expression is conserved between the hematopoietic lineages of mice and humans. We data mined two previously published scRNA seq datasets to compare the distribution of E-cadherin expression in HSPCs between mice and humans [35,36]. For extensive methodology comparison between mouse and human hematopoiesis by scRNA seq on HSPC populations we refer to Pellin et al. [36]. In both mice and humans, HSPC differentiation follows a hierarchical tree-like continuum of states, in which branches terminate in committed cell lineages [36]. Strikingly, the distribution of E-cadherin expression within the two-dimensional representation of the HSPC cell lineage is not conserved between mice and humans (Figure 4A,B). *CDH1* expression in human HSPCs selectively marks precursors of the erythroid lineage and is hardly expressed in the basophil/mast cell lineage (Figure 4A). In contrast, in mouse HSPCs, *Cdh1* is mostly expressed by precursors of the basophil/mast cell lineage, in agreement with our findings (Figure 4B). Overall, whereas E-cadherin expression is highly conserved in epithelia of mice and humans, the distribution of E-cadherin expression within the HSPC lineage is not consistently conserved. We additionally tested E-cadherin cell-surface expression on human basophil-enriched CD123^+^ HLA-DR^-^ IgE^+^ cell population derived from human peripheral blood mononuclear cells (PBMCs) (Figure 4C, Supplementary Figure S4A,B) [37]. However, we did not detect E-cadherin membrane protein expression on this enriched population of human basophils, in contrast to human erythroblasts, which we included as a positive control (Figure 4C, Supplementary Figure S4A–C). This corresponds with the human scRNAseq database, where E-cadherin mRNA is hardly detected in the basophil/mast cell lineage of HSPCs (Figure 4A). The prominent E-cadherin expression on basophil precursors and circulating basophils in mice is therefore not conserved in humans.

We also examined *CDH1* expression in 100 defined human leukemic cell lines using DSMZcellDive, an online tool from the Leibniz institute, and identified that the human leukemic cell line KU-812 displays high *CDH1* expression (Figure 4D) [32]. This is of particular interest as KU-812 cells have been characterized as displaying characteristics of late HPCs that are committed to the basophil lineage [38]. Intriguingly, early characterization of this cell line revealed that KU-812, as well as having basophil characteristics, also expressed erythroid defined markers, as defined by α-Globin and GYPA protein expression [38]. We confirmed these findings by examining the expression of erythroid markers (e.g., TFRC (encoding CD71), GYPA and HBA1 (encoding α-globin) using DSMZcellDive (Figure 4D,E). These findings are of particular interest as the basophil lineage separates prior erythroid lineage formation in both mice and humans (Figure 4A,B). KU-812 therefore may reflect transformed HPCs with bipotent lineage formation potential towards the basophil and erythroid cell lineages. Overall, the obtained results underscore a distinguished role for E-cadherin signaling in mouse basophils compared to human erythroid progenitors.

## 4. Discussion

In this study, we compared human with mouse hematopoiesis to examine whether mice can be used to investigate the function of E-cadherin in human hematopoiesis. We defined E-cadherin expression as mostly marking the basophil/mast cell lineage in murine BM, which is in agreement with previous findings [21,39]. E-cadherin expression also marks a small fraction of late myeloid HPCs that are able to reconstitute the myeloid and erythroid cell lineage in vitro under conditions that favor myeloid or erythroid outgrowth.

In contrast to that of the mouse, the human basophil lineage does not express detectable mRNA or protein levels of E-cadherin. E-cadherin, therefore, may display a mouse-specific regulator of basophil behavior, as also seen for Mcpt8, which also marks the basophil lineage in mouse but is absent in human basophils [28]. Alongside proteases, mouse and human basophils also display a distinct cytokine receptor profile, further underscoring the differences between basophil regulation in mice and humans [40,41,42].

Within the mouse basophil lineage, the molecular function of E-cadherin remains to be determined. However, despite the small sample size, we found E-cadherin not to be essential in driving basophil formation in mice. Instead, its function may underlie the immune response of basophils. Alongside homophilic interactions, E-cadherin also facilitates the formation of heterophilic interactions as described for T-cell and NK-cell associated ligands CD103 and KLRG1 [43,44,45,46,47,48,49,50,51]. E-cadherin-CD103/KLRG1 interactions mostly act as an anti-inflammatory regulatory signal and thereby may enable basophils to attenuate T-cell activity at the site of inflammation [49,50,52,53]. Additionally, basophils have been found to promote not only inflammatory responses, but also anti-inflammatory signals in the context of autoimmune disease, contact dermatitis and colitis [54,55,56]. Basophilic E-cadherin, therefore, may control the interplay of basophils with immune components to control their inflammatory response during inflammation. Future research should therefore address E-cadherin function under specific basophilic challenges such as parasite infections or allergies and regulation of its transcription.

Although we were unable to detect E-cadherin cell-surface expression on isolated human basophils from peripheral blood, human basophils may still upregulate E-cadherin expression at the site of inflammation. We identified that human-derived KU-812 cells, characterized as transformed late HPCs committed to differentiating the basophil lineage, also express E-cadherin as well as erythroid-defined proteins [38]. In mouse BM, we revealed that E-cadherin marks a subset of late HPCs that are also able to differentiate into the basophil lineage as well as into the erythroid lineage. Together these findings may imply that human BM likewise comprises a subset of late bifunctional HPCs that is marked by E-cadherin expression from which the leukemic KU-812 cell line upon transformation originated.

As well as distinguishing between the expression of E-cadherin in the basophilic lineages of mice and humans, our study also underscored the marked difference of E-cadherin expression within the erythroid lineages of mice and humans. Whereas E-cadherin mostly marks the erythroid lineage in human BM, its expression is absent within the erythroid lineage in mouse BM. More than two decades ago, human erythroblasts were already found to express E-cadherin, although, its regulation of erythropoiesis has never been resolved [22].

Whereas the regulation of mouse and human erythropoiesis is mostly resemblant under steady-state conditions, the homeostatic/erythropoietic response to anemia, also known as stress erythropoiesis, is differently controlled [57,58]. Stress erythropoiesis in mice is mainly driven by activating extramedullary erythropoiesis in the spleen; whereas, stress erythropoiesis in humans is predominantly (but not restrictively) activated in the BM [57,58]. Stress erythropoiesis in the mouse spleen is well-characterized, yet the molecular mechanisms underlying stress erythropoiesis in human BM are mostly elusive [59,60,61,62]. Although E-cadherin is not conserved in mouse splenic or BM erythroblasts, and neither controls stress erythropoiesis in mice, it may in fact involve the regulation of stress erythropoiesis in human BM, in which molecular regulation is mostly elusive.

Overall, hematopoietic E-cadherin reveals evolutionary discrepancies between mice and humans. This is also in line with a marked variation in transcriptome composition during terminal erythropoiesis between mice and humans [63,64]. One could therefore question if the mouse can be a representative model for human hematopoietic E-cadherin function and more general human erythropoiesis. This, therefore, also calls into question to what extent alternative animal models (e.g., zebrafish, rat, rabbit or monkey) can be used to model E-cadherin signaling in human erythropoiesis. Rats, for example, in contrast to mice, mimic stress erythropoiesis that occurs in humans by strongly expanding erythropoiesis in their BM [57].

Furthermore, it remains to be defined by what means E-cadherin expression is differently regulated in hematopoiesis in humans and mice. Our evaluation of defined regulators of E-cadherin expression (e.g., *SOX2*, *ESR2*, *TCF7*, *GATA-2*, *GATA-3*, *GATA-4*, *SMARCA4*, *SMAD3*, *SOX9*, *Runx1*, *EOMES*, *PPAR*), by data mining single-cell RNA-seq profiles of mouse and human hematopoiesis, did not reveal distinct expression patterns [36]. Yet, erythroid-defined transcription factors have been identified as displaying an altered chromatin occupancy in mice and humans [65]. This thereby implies that, even when expression levels of transcription regulators in mouse and human erythropoiesis are similar, their transcriptional outcome may differ. This could also explain why E-cadherin expression is not conserved within the human basophil lineage that, similarly to mice, is characterized by high GATA-2 expression, which is implicated in driving E-cadherin expression in the mouse basophil lineage [21]. Differences in E-cadherin expression between human and mouse hematopoiesis can also be induced by differences in promoter methylation, as methylation of the promoter region of *CDH1* is well-known to downregulate E-cadherin expression [66,67].

Overall, we conclude that hematopoietic E-cadherin harbors both evolutionary conserved and species-specific functions for which models need to be carefully chosen.

## Data Availability

We will deposit our raw single-cell RNAseq datasets in publicly available repositories. The datasets and materials analyzed during the current study are available from the corresponding author upon reasonable request.

## Supplementary Materials

The following supporting information can be downloaded at: https://www.mdpi.com/article/10.3390/biom12111706/s1, Supplementary Figure S1: (A–D) Representative FACS gating strategy used to characterize E-cadherin^+^ bone marrow (BM) cells in *VaviCre;mTmG* mice, which is used in Figure 1B–F. (E) FACS analysis, following gating strategy (Supplementary Figure S1A–D) of CD45 coexpression with GFP fluorescence by BM cells derived from *VaviCre;mTmG* mice. (F) Representative gating strategy, following Supplementary Figure S1A–D of basophils derived from *VaviCre;mTmG* BM. (G) Representative FACS analysis, of E-cadherin cell-surface expression within the BM basophil gate compared to live singlets derived from *VaviCre;mTmG* mice (depicted in Supplementary Figure S1F). (H) Representative FACS plot of *VaviCre;mTmG* BM stained with an isotype control antibody instead to determine background signal for the anti-E-cadherin antibody. (I) Representative FACS plots of unstained negative control samples on which gating strategy depicted in Supplementary Figure S1A–D and F are based. (I) Representative FACS analysis, following gating strategy described in Supplementary Figure S1A–D,F, of E-cadherin cell-surface expression and GFP fluorescence within the live singlet, non-ery and LIN^−^ gates by BM cells derived from *VaviCre;mTmG* and *VaviCre;Cdh1^F/F^;mTmG* mice as negative control. Supplementary Figure S2: (A) Representative FACS gates, following gating strategy described in Supplementary Figure S1A–D, to sort E-cadherin^+^;cKIT^+^ and E-cadherin^+^;cKIT^−^ bone marrow (BM) cells from *VaviCre;mTmG* mice used in Figure 2A–I. (B,C) Representative FACS gates, following gating strategy described in Supplementary Figure 1A–D, to sort E-cadherin^+^;cKIT^+^ and E-cadherin^+^; cKIT^−^ BM cells from *VaviCre*;*mTmG* mice, used in Figure 2J–L. (D,E) Representative FACS gate, following gating strategy described in Supplementary Figure S1A–D, to sort LIN^−^;cKIT^+^ BM cells from *VaviCre*;*mTmG* mice used in Figure 2J–L. (F) TFRC gene expression plotted for all t-SNE clusters of E-cadherin^+^ mouse BM cells as depicted in Figure 2H. Supplementary Figure S3: Representative FACS analysis of IgE^+^;B220^−^ gated cells for cKIT cell-surface expression to examine the presence of cKIT-marked mast cells in *VaviCre*;*mTmG* (black graphs) and *VaviCre*;*Cdh1^F/F^;mTmG* mice (red graphs), depicted in histograms for (A) bone marrow (BM) (B) spleen (C) blood and (D) lung (Count Modal (A.U). (E,F) Representative FACS gate to define erythroblast population in Q2 in *VaviCre*;*mTmG* and *VaviCre;Cdh1^F/F^;mTmG* mice. (G) Quantification of CD71^+^;TER119^+^ erythroblasts in *VaviCre*;*mTmG* (black) and *VaviCre*;*Cdh1^F/F^;mTmG* (red) BM and (H) spleen. Representative FACS analysis to define E-cadherin cell-surface expression on CD71^+^;TER119^+^ mouse erythroblasts in BM (I) and spleen (J) in *VaviCre*;*mTmG* (black) and *VaviCre*;*Cdh1^F/F^;mTmG* (red) mice depicted as histograms. BM-derived basophils (blue) were used as a positive control for E-cadherin cell surface staining. (L) Red blood cell (RBC) count (RBCs/Liter), (M) hemoglobin (mmol/L) and (N) hematocrit (volume %), measured in blood of *VaviCre*;*mTmG* (black dots, *n* = 8 animals) and *VaviCre;Cdh1F/F;mTmG* mice (red dots, *n* = 5 animals) in steady state versus eight days after LCMV infection. Supplementary Figure S4: (A,B) Representative FACS gate to enrich for basophils in human peripheral blood mononucleated cells using CD123, HLA-DR and IgE as used in Figure 4C. (C) Representative FACS gate to enrich for CD71^+^; CD235a^+^ human erythroblasts as used in Figure 4C as a positive control for E-cadherin cell-surface expression. Supplementary Table S1: Flowcytometry antibodies used on mouse tissue. Supplementary Table S2: Flowcytometry antibodies used on human tissue.
