# Supplementary material for: E-Cadherin Expression Distinguishes Mouse from Human Hematopoiesis in the Basophil and Erythroid Lineages
Source: Biomolecules. 2022 Nov 17;12(11):1706. doi: 10.3390/biom12111706 (PMC9688100; doi:10.3390/biom12111706)
Supplement: Supplementary file 1 [file biomolecules-12-01706-s001.zip › Supplemental Tables.pdf]

Supplemental Table S1. Flow cytometry antibodies used on mouse tissue

Target	Clone	Conjugate	Manufacturer	Catalog no.
**B220**	RA3-6B2	APC	eBioscience	17-0452-82
**B220 (LIN)**	RA3-6B2	PE	Biolegend	103207
**B220 (LIN)**	RA3-6B2	AF488	eBioscience	53-0452-82
**CD11b (LIN)**	M1/70	PE	Biolegend	101207
**CD11b (LIN)**	M1/70	AF488	Biolegend	101219
**CD31**	390	BV421	Biolegend	102423
**CD31/PECAM-1**	390	AF-647	Biolegend	102415
**CD4 (LIN)**	GK1.5	PE	eBioscience	12-0041-83
**CD4 (LIN)**	GK1.5	FITC	eBioscience	11-0041-85
**CD45**	Ly5	PE	BD Biosciences	553081
**CD71**	RI7217	BV421	Biolegend	113813
**CD8 (LIN)**	53-6.7	PE	eBioscience	11-0081-86
**CD8 (LIN)**	53-6.7	FITC	eBioscience	11-0081-82
**cKIT**	2B8	APC	eBioscience	17-1171-83
**E-cadherin**	DECMA-1	PE-Cy7	Biolegend	147310
**E-cadherin**	DECMA-1	AF488	eBioscience	53-3249-82
**E-cadherin**	DECMA-1	EF-660	eBioscience	50-3249-80
**FcER-1**	MAR-1	PE	eBioscience	12-5898-82
**IgE**	R35-72	BUV395	BD Biosciences	744283
**IgE**	RME-1	PE	Biolegend	406907
**LY-6G/C (LIN)**	RB6-8C5	PE	eBioscience	12-5931-82
**LY-6G/C (LIN)**	RB6-8C5	AF488	Biolegend	108417
**Sca-1**	D7	BV786	Biolegend	108139
**TER119**	TER119	APC	Biolegend	116211
**TER119**	TER119	Percp CY5.5	eBioscience	45-5921-80
**TER119 (LIN)**	TER119	AF-488	Biolegend	116215
**Rat IgG1, Isotype Ctrl**	RTK2071	PE-Cy7	Biolegend	400415

Supplemental Table S2. Flow cytometry antibodies used on human tissue

Target	Clone	Conjugate	Manufacturer	Catalog no.
**CD123**	AC145	FITC	Miltenyi	130-090-897
**CD235**	REA175	APC	Miltenyi	130-188-356
**CD235**	JC159	FITC	Origene	DM066F
**CD235**	JC159	PE	Acris	DM066R
**CD71**	AC102	VioBlue	Miltenyi	130-101-627
**E-cadherin**	67A4	Af488	BD Biosciences	563570
**HLA-DR**	G46-6	PE	BD Biosciences	555812
**IgE**	G7-26	BUV395	BD Biosciences	744321
**Mouse IgG1k isotype ctrl**	MOPC-21	PE-CY7	Biolegend	400126
