# Supplementary figures and images for: E-Cadherin Expression Distinguishes Mouse from Human Hematopoiesis in the Basophil and Erythroid Lineages
Source: Biomolecules. 2022 Nov 17;12(11):1706. doi: 10.3390/biom12111706 (PMC9688100; doi:10.3390/biom12111706)

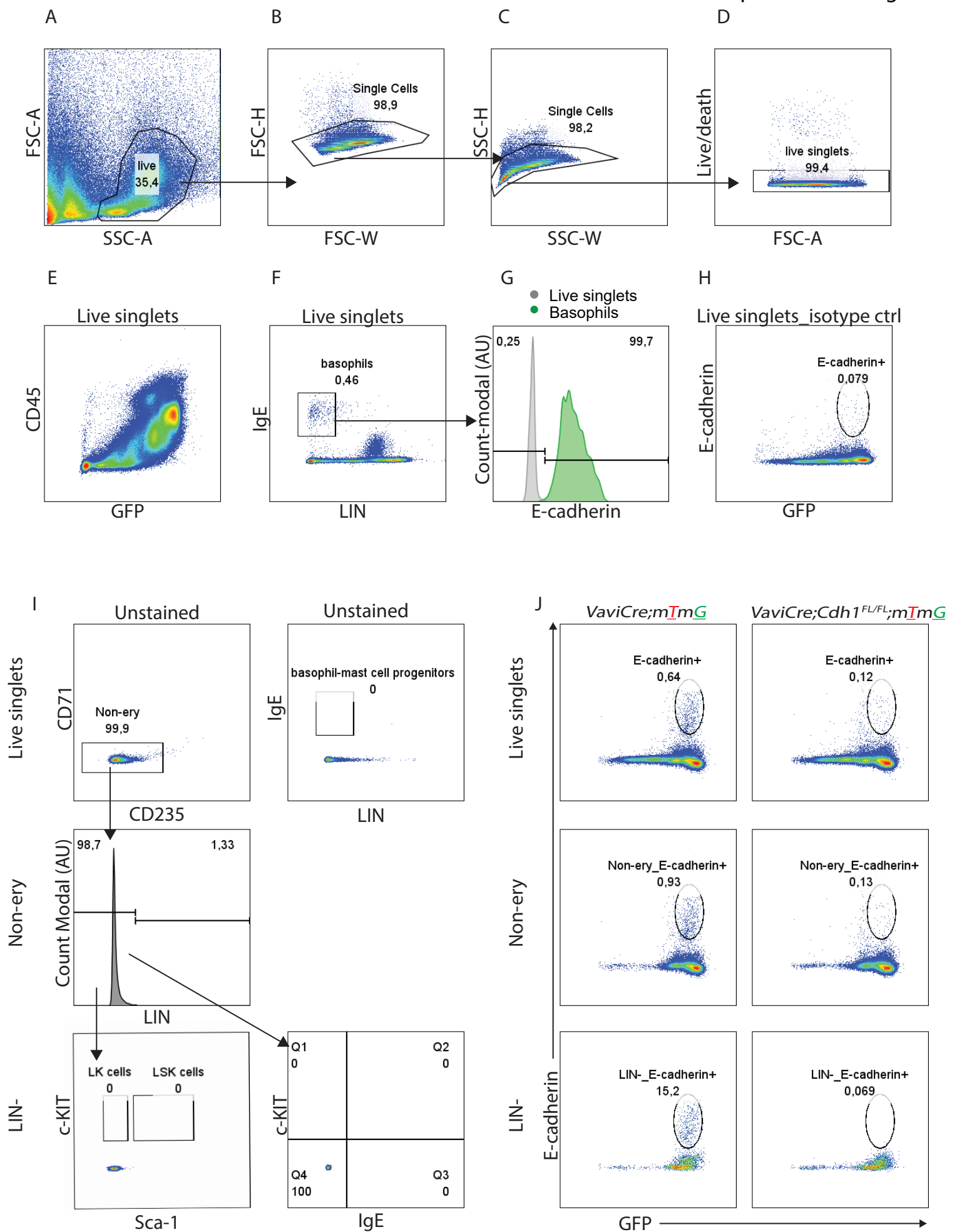

Supplement: Supplementary file 1 [file biomolecules-12-01706-s001.zip › Krimpenfort et al., Figure S1.pdf]

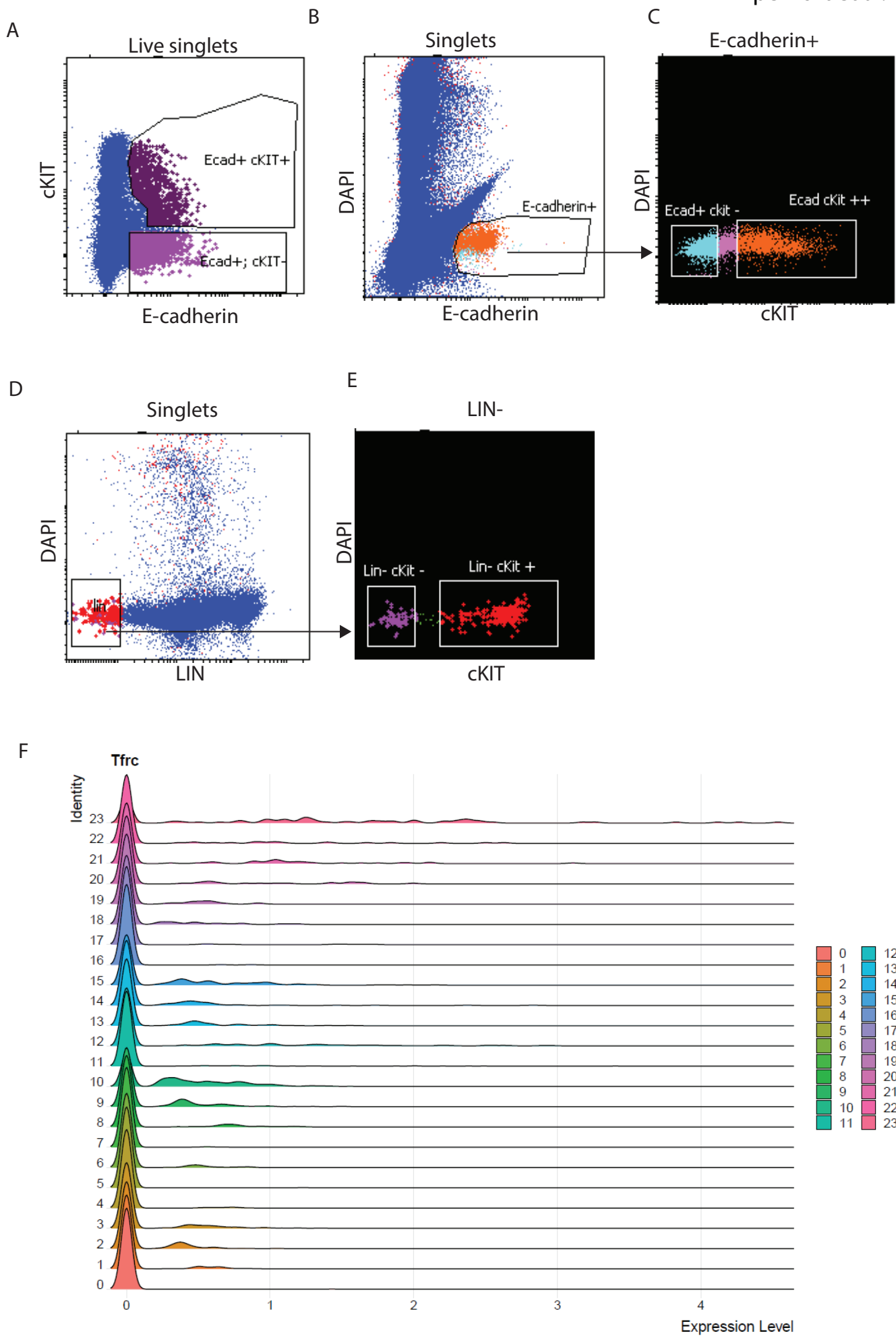

Supplement: Supplementary file 1 [file biomolecules-12-01706-s001.zip › Krimpenfort et al., Figure S2.pdf]

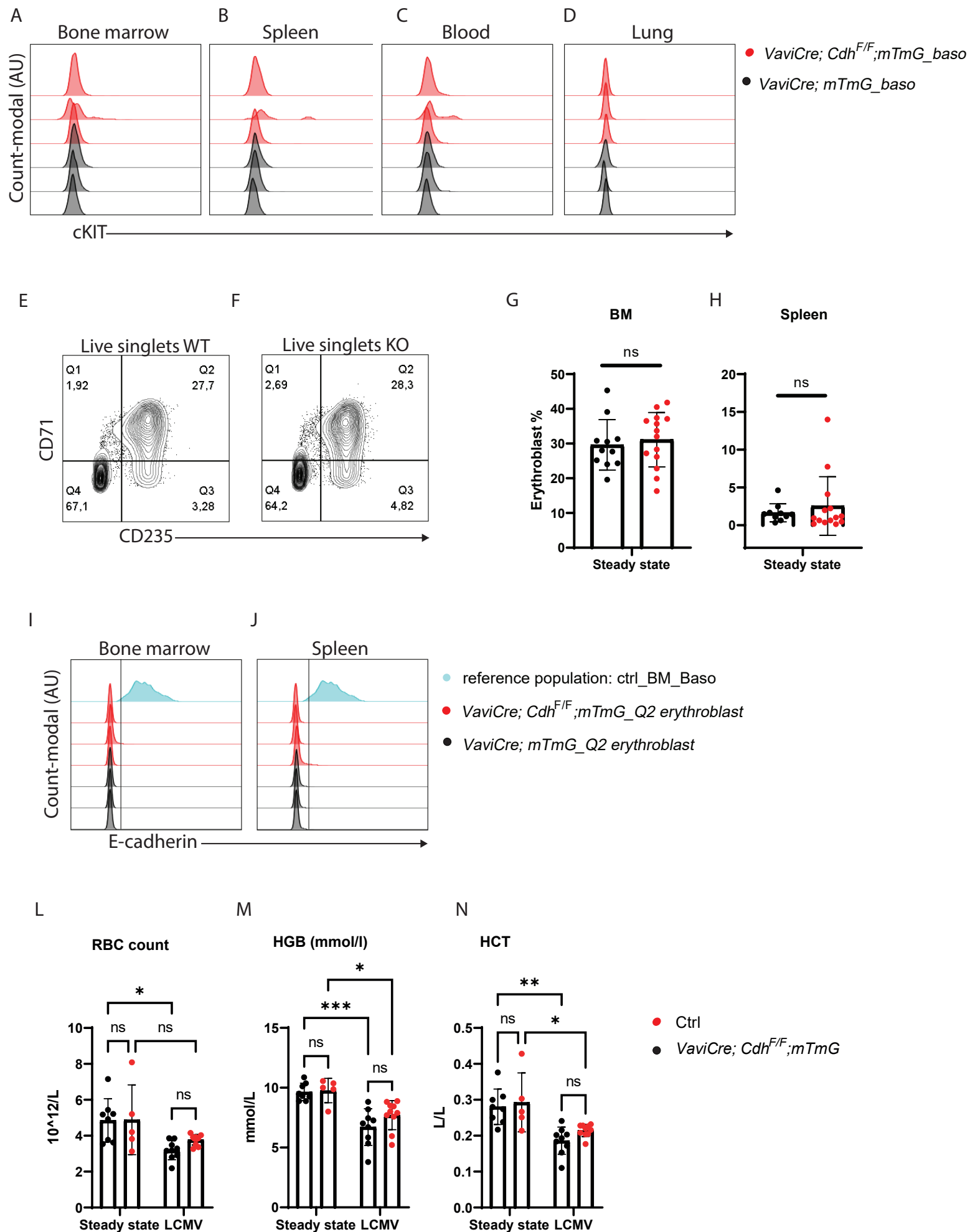

Supplement: Supplementary file 1 [file biomolecules-12-01706-s001.zip › Krimpenfort et al., Figure S3.pdf]

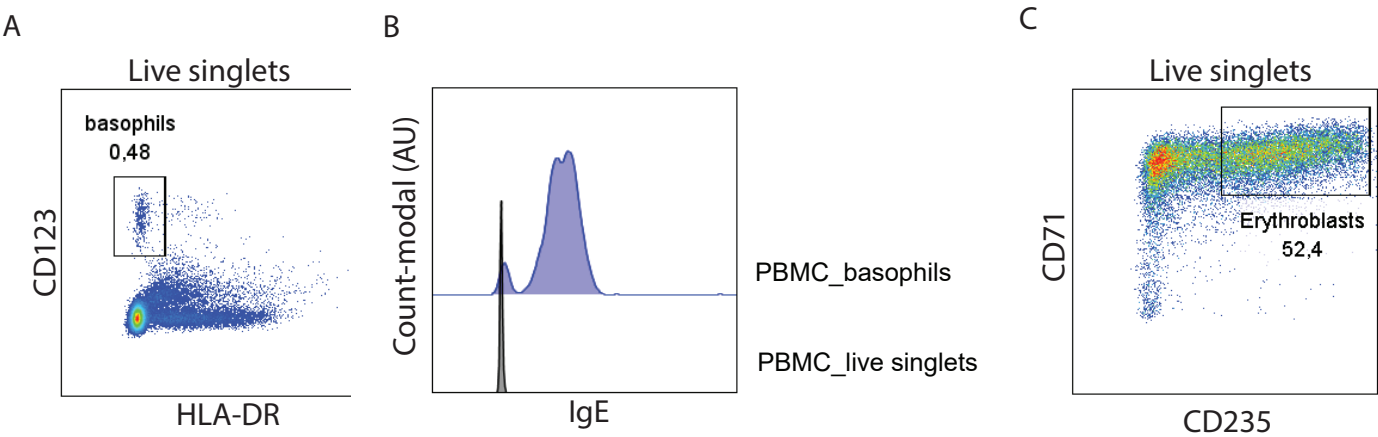

Supplement: Supplementary file 1 [file biomolecules-12-01706-s001.zip › Krimpenfort et al., Figure S4.pdf]
